# Special Issue: From Host–Pathogen Interaction to Host-Directed Therapies

**DOI:** 10.3390/microorganisms9122606

**Published:** 2021-12-17

**Authors:** Maria Salomé Gomes, Luisa Pereira

**Affiliations:** 1 i3S—Instituto de Investigação e Inovação em Saúde, Universidade do Porto, 4200-135 Porto, Portugal; 2 IBMC—Instituto de Biologia Molecular e Celular, Universidade do Porto, 4200-135 Porto, Portugal; 3 ICBAS—Instituto de Ciências Biomédicas Abel Salazar, Universidade do Porto, 4050-313 Porto, Portugal; 4 IPATIMUP—Instituto de Patologia e Imunologia Molecular, Universidade do Porto, 4200-135 Porto, Portugal

Despite the enormous progress made in the last few decades, infectious diseases still represent a huge challenge to human society and health systems, as evidenced by the recent SARS-CoV-2 pandemic. Studies on host–pathogen interactions have been key in the identification of the most important determinants of infection outcome. From the pathogen side, these studies contribute to the identification of virulence factors, which may be targeted by anti-virulence strategies, less prone to resistance development than anti-viability agents. From the host side, these studies identify those aspects of host metabolic and homeostatic status that have the highest impact on resistance or susceptibility to infection. In this context, it is important to note that high-throughput omics studies represent a powerful approach to reveal diverse and subtle impacts of infection on host health status, both immediate and life-long, that are not otherwise anticipated. The identification of host targets of infection with a role in disease progression or resistance represents a new opportunity to envisage host-directed therapies. In a time when antibiotic treatments are being challenged by increasing bacterial resistance rates, host-directed therapies represent an appealing complementary strategy to combat infectious diseases. The Special Issue “From Host–Pathogen Interaction to Host-Directed Therapies” includes eight research papers, one brief report and two reviews dealing with different human pathogens and how they interact with their host.

The work by Pérez-Arques et al. [1] is dedicated to the study of virulence factors in Mucolares, an emerging group of fungal pathogens associated with high lethality and treatment failure. The authors investigated the role of white-collar genes in the virulence potential of *Mucor lusitanicus*. White-collar genes are best known for their role in regulating light-dependent responses, while their role in virulence has not been thoroughly explored before. In this work, the authors used an integrated transcriptomic and functional approach to study the participation of mcwc-1a, mcwc-1b, and mcwc-1c in host–pathogen interactions. Their results revealed mcwc-1a as a master regulator controlling an extensive gene network, involved in several key functions such as cell motility and cytoskeleton rearrangements, as well as oxidative response in the context of the interaction with the host phagocytic cells. Interestingly, this experimental approach allowed the detection of several genes of “unknown function”. Their future study may reveal promising new targets for the development of antifungal compounds. 

Highlighting the binomial contribution of microbe virulence and host resistance to severe infection, the paper by Pedro et al. [2] reported an interesting COVID-19 case study. A 17-year-old Portuguese female presented a clinically severe and prolonged (97 days) viral shedding disease. The authors obtained the whole viral genome and screened a human genome-wide array (1 million variants) on seven time-spanned samples from the patient. The results were surprising for the pathogen counterpart: at diagnosis, the patient (and her mother) was infected by a SARS-CoV-2 affiliated with the 20A lineage; nine days later, a second 20B lineage (diverging by six variants) had 3% frequency; two months later, only the 20B was present. The patient had polygenic risk scores for hospitalization and severe respiratory disease within the normal distributions for a Portuguese population cohort. This important paper is the first same-species co-infection case reported for SARS-CoV-2, and it reinforces the importance of patient isolation and/or ventilation/air renovation of hospital shared spaces, in order to avoid reinfection of patients whose immune systems is already fighting a viral variant. 

If same-species co-infection is of concern in clinical terms, of course different-species co-infection is another important factor to take into account. The paper by Malagnino et al. [3] focused on evaluating whether the presence of anti-hepatitis B core antibodies (HBcAb positivity) could influence the control of human immunodeficiency virus (HIV) viremia in patients living with HIV (PLWH) who switch to a two-drug antiretroviral therapy containing lamivudine (2DR-3TC). The analysis of the retrospective observational multicenter study led the authors to conclude that the condition of potential occult HBV infection should be carefully considered when selecting PLWH candidates for 2DR switching, as they are potentially at risk of possible future therapeutic failure.

The paper by Rouzine [4] also dealt with the host–pathogen binomial in defining the outcome of HIV infection. The author developed a mechanistic mathematical model to predict the speed of progression to acquired immunodeficiency syndrome (AIDS) in untreated and treated (suboptimal therapy) patients, based on a single-time measurement of several virological and immunological parameters. The author tested and validated a model of AIDS progression based on the virus adaptation that fits the existing data better than the standard homeostatic deregulation-based models. In fact, the evolution model agrees with the observed slow, negative correlation between the time to AIDS and viremia, and the positive correlation observed between the speed of the progression to AIDS and the level of the immune activation.

Turning to the host arm of the equation, three papers clearly illustrate the role played by the host’s ancestral background on the differential susceptibility/resistance to pathogens within the human species. The paper by Cavadas et al. [5] characterized the gut microbiome from RNASeq data obtained on stomach samples of non-disease and gastric cancer patients of European and Asian ancestries, and available in public databases. Despite the decrease in microbiome diversity observed in the disease status, the authors were able to verify that it mimicked host diversity across the world, with European gastric cancer microbiome profiles clustering together, distinct from Asian ones. They further confirmed that this parallel host–bacteria population structure could be in part explained by host genetic variants that display frequency heterogeneity between population groups. These 16 microbiome quantitative trait loci were mainly linked to the immune system or cellular features that may play a role in enabling microbe colonization and inflammation. The authors pursued this topic in another paper in the current Special Issue [6] by conducting the first in vitro coinfection assays with dual human- and bacterium-ancestries, from African and European backgrounds. They used *Helicobacter pylori* in these experiments, which has been recognized as a class 1 gastric carcinogen. The genome wide gene expression evaluation of the host response to *H. pylori* infection showed high variability due to human ancestral background, especially on genes related with innate immune system, metabolism and cell homeostasis. Interestingly, the African human ancestry displayed signs of coevolution with *H. pylori*, while the European ancestry appeared to be maladapted, compatible with a longer co-existence in the African continent relatively to the more recent disruptive dual bottleneck of the host and pathogen at the out-of-Africa migration. The inclusion of the host ancestry factor in the evaluation of biomarker association with infectious diseases has the additional value of potentially explaining several non-reproducible results. This was the case reported by Rito et al. [7] for the rs17525495 variant in the Leukotriene A4 hydrolase, previously linked to tuberculosis susceptibility and outcome in Vietnam. The authors characterized this and other 112 neighbor variants in a Portuguese dataset, and concluded that the variants low frequencies in the European cohort render them less informative in terms of public health in this continent, in relation to Asia. Another study dealing with host susceptibility to tuberculosis (TB) is that by Matos et al. [8]. In this study, the authors showed an increased expression of the sialylated glycan structure Sialyl-Lewis X (SLeX) in the mouse lung epithelium upon *M. tuberculosis* infection. This increase in the SLeX glycan epitope is accompanied by an altered lung tissue transcriptomic signature, with up-regulation of genes coding enzymes that are involved in the SLeX core-2 O-glycans biosynthetic pathway. Glycans display increasingly recognized roles in pathological contexts; however, their impact in the host–pathogen interplay in many infectious diseases remains largely unknown. This study provides novel insights into a possible contribution of glycosylation to shape TB disease outcome.

Immune activation during infection has a broad impact in several homeostatic circuits in the host, as seen in the well-known example of iron metabolism. Ferritin, first discovered as an iron storage protein, has emerged as an important disease biomarker in inflammatory diseases, or diseases in which inflammation has a central role such as infection. Moreira et al. [9] reviewed recent research on ferritin functions, as well as its role in diseases with an inflammatory component and its potential as a target in host-directed therapies. Oliveira et al. [10], on the other hand, reviewed how immune mediators produced in response to infection may dysregulate the deposition of mineral matrix by osteoblasts and/or the resorption of bone by osteoclasts. Bone loss pathologies often develop in response to infection, and their detection and treatment are challenging. Therefore, further understanding of the impact of infections on bone metabolism is imperative for the early detection, prevention, and/or reversion of bone loss. 

The paper by Heimesaat et al. [11] represented one clear example of host-directed therapy with promising results against an important emerging pathogen. *Campylobacter jejuni* causes frequent and serious infections in humans and multi-drug resistant isolates are emerging worldwide. Antibiotics-independent approaches to treat these infections are therefore highly desirable. In this study, the authors used a mouse model to test the effect of the natural product cardamom essential oil to treat acute campylobacteriosis. The treatment resulted in lower intestinal pathogen loads and improved clinical outcome, with less inflammatory sequelae. The authors suggested that cardamom essential oil represents a promising therapeutic tool for the combat of acute campylobacteriosis, including the prevention of post-infectious morbidities.

Altogether, this Special Issue “From Host–Pathogen Interaction to Host-Directed Therapies” reunites a diverse set of studies addressing this fascinating topic. Valuable new data on pathogen virulence factors, host susceptibility/resistant traits and new models to integrate these factors into disease progression prediction were reported. The diversity of experimental approaches, from genomics and transcriptomics to animal infection models, will certainly inspire future studies in this field.
